# Point-of-Care EEG for Non-Convulsive Seizure and Status Epilepticus: Advances, Limitations, and Future Directions

**DOI:** 10.3390/jcm15041643

**Published:** 2026-02-22

**Authors:** Ana Leticia Fornari Caprara, Jamir Pitton Rissardo, Hana Rababeh, April Pivonka, Priya Shah, Kaitlyn Piotrowski, Matthew George Petruncio, Anusha Keshireddy, Zehra Jaffri, Arthur Gribachov, Ruchika Moturi, Haashim Khurram, Manisha Koneru, Evren Burakgazi-Dalkilic

**Affiliations:** 1 Neurology Department, Cooper University Hospital, Camden, NJ 08103, USA; gribachov-arthur@cooperhealth.edu (A.G.); mkoneru2@alumni.jh.edu (M.K.); burakgazi-dalkilic-evren@cooperhealth.edu (E.B.-D.); 2 Department of Neurology, University of Michigan, Ann Arbor, MI 48104, USA; hrababeh@umich.edu; 3 Department of Neurology, Cooper Medical School of Rowan University, Camden, NJ 08103, USA; pivonk79@rowan.edu (A.P.); shahpr67@rowan.edu (P.S.); piotro76@rowan.edu (K.P.); petruncim2@rowan.edu (M.G.P.); keshir72@rowan.edu (A.K.); moturi37@rowan.edu (R.M.); khurra74@rowan.edu (H.K.); 4 Department of Neurology, Rowan-Virtua School of Osteopathic Medicine, Stratford, NJ 08084, USA; jaffri42@rowan.edu

**Keywords:** point-of-care EEG, non-convulsive status epilepticus, non-convulsive seizures, stroke, traumatic brain injury, delirium, artificial intelligence, accuracy, cost-effectiveness

## Abstract

Point-of-care electroencephalography (POC-EEG) has emerged as a practical tool for the rapid detection of non-convulsive seizures (NCS) and non-convulsive status epilepticus (NCSE) in acute neurological settings where access to conventional EEG is often delayed. This narrative review synthesizes current evidence on the clinical applications, tech-no-logical evolution, and limitations of POC-EEG systems across adult and pediatric populations. Available data suggest that POC-EEG is associated with earlier seizure identification, more timely antiseizure treatment decisions, and reduced dependence on inter-facility transfers in selected healthcare settings. Beyond seizure detection, POC-EEG has shown potential utility in the assessment of acute encephalopathy due to conditions such as stroke, traumatic brain injury, delirium, and post-cardiac arrest states. Recent advances in device portability and artificial intelligence-assisted interpretation have expanded accessibility, enabling use by non-specialist clinicians; however, reduced spatial resolution, artifact susceptibility, and variable performance in focal or low-burden epileptiform activity remain important limitations. Automated detection algorithms show high accuracy for sustained seizure burden but require cautious interpretation and further prospective validation. Ethical and health-system considerations, including equitable access, diagnostic stewardship, and data governance, are increasingly relevant as adoption grows. Overall, POC-EEG represents a promising adjunct to conventional EEG that may improve early diagnostic workflows in acute neurological care, while definitive impacts on long-term outcomes warrant further study.

## 1. Introduction

Epilepsy is characterized by recurrent, unprovoked seizures resulting from abnormal, excessive, or synchronous neuronal activity within the brain. Individuals with epilepsy have a threefold increased risk of premature mortality compared to the general population [1]. According to the 2021 Global Burden of Disease study, approximately 52 million people worldwide are living with active epilepsy, with more than 80% residing in low- and middle-income countries [2].

The International League Against Epilepsy (ILAE) defines status epilepticus as a condition arising either from the failure of mechanisms responsible for seizure termination or from the initiation of mechanisms that lead to abnormally prolonged seizures [3]. In convulsive status epilepticus, the threshold for abnormally prolonged seizure activity is five minutes. Non-convulsive status epilepticus does not have a single universally accepted time cutoff. Conceptual definitions of prolonged seizures proposed by the ILAE emphasize seizure duration and risk of neuronal injury in a seizure-type-specific manner, whereas EEG-based definitions rely on electrographic features and seizure-burden criteria operationalized by the ACNS (see Table 1: Operational Definitions Used in This Review). The American Clinical Neurophysiology Society (ACNS) describes the concept of NCSE as ictal activity constituting more than 20 percent of an hour of recording (i.e., more than 12 min). Studies have demonstrated that neurologic decline increases substantially if the maximal hourly seizure burden (SzB) exceeds 20% [4].

Current estimates suggest that 30–50% of patients with status epilepticus (SE) subsequently develop epilepsy, although robust evidence-based studies are limited, and the relationship between epilepsy and SE warrants further investigation [5]. The annual incidence of SE in the United States is estimated at 15–20 cases per 100,000, with approximately 63% of these cases classified as NCSE [6]. In a study utilizing electroencephalographic monitoring in the ED, about 37% of individuals who underwent EEG for acute encephalopathy were diagnosed with NCSE [7].

Because patients with NCSE typically present to ED and medical intensive care units, early identification is often hindered by factors such as highly variable clinical presentations, challenging EEG patterns, and limited availability of EEG machines, technicians, and EEG-trained providers. Delayed diagnosis poses significant risks, including postponed treatment for toxic, infectious, and metabolic causes of acute encephalopathy, undertreatment of seizures—which increases the risk of refractory and super-refractory SE—prolonged intubation, and extended hospitalization [8].

The risk of neuronal injury and poor clinical outcome in non-convulsive status epilepticus increases with longer electrographic seizure duration and higher seizure burden, but varies substantially according to seizure etiology, patient susceptibility, and the surrounding clinical context. Unlike convulsive status epilepticus, a clearly defined ILAE t2 threshold for irreversible injury in NCSE has not been established, and outcomes cannot be predicted based on seizure duration alone. Clinical outcomes in NCSE reflect the interaction of seizure burden, duration, etiology, and host vulnerability rather than a single time-based threshold [9].

Experimental and translational data suggest that prolonged seizure activity is associated with time-dependent synaptic receptor trafficking, including internalization of GABA_A_ receptors and reduced inhibitory efficacy, which contributes to early pharmacoresistance to benzodiazepines. This biological progression supports early EEG-guided identification of ongoing ictal activity and consideration of timely combination therapy in delayed, treatment-naïve presentations of status epilepticus [8,9,10].

EEG remains an essential tool for diagnosing epilepsy, including NCSE and NCS. However, while EEG provides valuable diagnostic information, conventional recording methods have limitations: they are often costly, time-consuming, and uncomfortable for patients, which may delay timely diagnosis and treatment [10].

Non-convulsive status epilepticus is frequently diagnosed late because clinical manifestations are subtle or nonspecific, and definitive diagnosis depends on EEG confirmation. In many cases, patients are first evaluated hours after seizure onset and may remain treatment-naïve despite ongoing ictal activity, particularly when access to conventional EEG is delayed. Recent conceptual frameworks describing early pharmacoresistance in status epilepticus emphasize this scenario—ongoing seizures with delayed diagnosis and delayed initiation of therapy—as a high-risk state in which outcomes may worsen due to time alone [11,12].

In this setting, prompt access to EEG becomes critical. Point-of-care EEG addresses this diagnostic gap by enabling early bedside confirmation or exclusion of ongoing seizures, thereby supporting timely guideline-concordant therapy when seizures are present and preventing unnecessary treatment escalation when they are not.

In this context, point-of-care EEG (POC-EEG) has emerged as a vital tool in resource-limited settings, enabling timely and cost-effective management of patients with suspected NCSE. The aim of this manuscript is to provide a comprehensive literature review assessing the use of POC-EEG in the diagnosis of NCS and NCSE, evaluating its diagnostic performance compared to standard EEG, and discussing its limitations and potential role in future clinical practice.

**Table 1:** Operational Definitions Used in This Review.

Domain	Definition	How Used in This Review
ILAE (2015) conceptual framework	The International League Against Epilepsy defines status epilepticus using two time points: t1, the time beyond which a seizure is unlikely to stop spontaneously, and t2, the time beyond which there is a risk of long-term neuronal injury. These thresholds are conceptual, vary by seizure type and semiology, and are not intended as strict EEG or treatment cutoffs [3].	Used to frame the pathophysiology and clinical urgency of prolonged seizures. Not used as a rigid diagnostic threshold for NCSE.
ACNS EEG terminology	The American Clinical Neurophysiology Society defines electrographic seizures, electrographic status epilepticus, and seizure burden based on EEG features. Electrographic status epilepticus is typically operationalized as ≥10 min of continuous ictal activity or seizures occupying ≥20% of an hour of recording [13].	Used for operational EEG interpretation, comparison across studies, and discussion of POC-EEG performance and AI-based seizure-burden metrics.
Seizure burden	Proportion of time occupied by electrographic seizures over a defined EEG epoch (e.g., minutes per hour). Higher seizure burden correlates with worse neurological outcomes [14].	Used to describe risk stratification, AI-assisted detection, and clinical decision-making rather than to redefine SE time thresholds.
Ictal–interictal continuum (IIC)	EEG patterns with rhythmic or periodic features that fall between clearly interictal and clearly ictal activity. These patterns may or may not represent ongoing seizures and often require clinical correlation [13].	Discussed separately from confirmed electrographic seizures. IIC patterns are not automatically classified as NCSE and are interpreted in conjunction with clinical context, evolution, and response to treatment.

## 2. Methodology

This narrative review was conducted to synthesize current evidence regarding the clinical utility, technological advancements, limitations, and future directions of POC-EEG in the detection of NCS and NCSE. Relevant literature published in English from January 2015 to October 2025 was identified through searches of PubMed, Embase, and Google Scholar using combinations of the following keywords: “point-of-care EEG,” “rapid EEG,” “non-convulsive seizures,” “non-convulsive status epilepticus,” “acute neurological care,” “stroke,” “traumatic brain injury,” “delirium,” “artificial intelligence,” and “cost-effectiveness.” Additional sources were identified by screening the reference lists of selected articles and recent guidelines from professional societies.

Throughout this review, we explicitly distinguish between conceptual, time-based definitions of status epilepticus proposed by the International League Against Epilepsy (ILAE) and operational EEG-based criteria defined by the American Clinical Neurophysiology Society (ACNS), including electrographic seizures, electrographic status epilepticus, and seizure-burden thresholds (see Table 1: Operational Definitions Used in This Review).

Eligible sources included original research studies, systematic and narrative reviews, clinical trials, validation studies of POC-EEG devices, consensus statements, and professional society guidelines relevant to acute neurological care. Both adult and pediatric studies were considered. Case reports and small case series were included selectively when they provided unique insights into feasibility, workflow integration, or limitations of POC-EEG technology.

Article selection was guided by relevance to the clinical applications of POC-EEG rather than by predefined outcome measures. References of included articles were manually screened to identify additional pertinent publications. Evidence was synthesized qualitatively and organized thematically, with emphasis on diagnostic performance, clinical workflow, seizure-burden assessment, artificial intelligence-assisted interpretation, and health-system considerations.

Given the narrative nature of the review and the heterogeneity of study designs and outcomes, no formal meta-analysis or quantitative risk-of-bias assessment was performed. The intent of this review is to provide a clinically oriented overview of the evolving role of POC-EEG rather than a comprehensive systematic evaluation of effect sizes.

The review emphasizes recent advances in device technology, integration of artificial intelligence, and the evolving role of POC-EEG in emergency and critical care settings. Limitations of the current literature, including heterogeneity in study design and outcome measures, are discussed.

## 3. Types of POC-EEG

Broadly, POC-EEG platforms can be categorized by their electrode montage configuration, intended clinical environment, and degree of automation. Table 2 provides a comparative overview of commercially available POC-EEG systems (Table 2).

EEG patterns along the ictal–interictal continuum (IIC), including rhythmic or periodic discharges without clear evolution, represent a diagnostic gray zone between interictal and ictal activity. In this review, such patterns are discussed separately from confirmed electrographic seizures and are not automatically classified as non-convulsive status epilepticus. Interpretation of IIC patterns requires clinical correlation, assessment of temporal evolution, and, in some cases, evaluation of treatment response. POC-EEG may facilitate early recognition of these patterns, but definitive classification often requires expert review and longitudinal EEG assessment.

Regulatory status reflects clearance for EEG signal acquisition as reported in the literature and does not imply diagnostic accuracy, clinical outcome benefit, or equivalence to conventional EEG. Economic and reimbursement considerations vary by institution and jurisdiction and are therefore not summarized as universal claims. This table is intended for comparative and educational purposes only and should not be interpreted as an endorsement of any specific device.

Conventional EEG technology employs a full montage setup, with electrodes applied in the standard 10–20 configuration using at least 19 channels [15]. However, this process is time-consuming and typically requires specialized EEG technologists. In contrast, most current POC-EEG systems use reduced montages with 10 or fewer electrodes. This reduced complexity enables minimal training requirements, simplified setup, and faster deployment. For example, Ceribell^®^ consists of a headband with integrated electrodes attached to a small portable EEG recorder and a built-in gel application system, while ZetoEEG^®^ uses dry electrodes. Ceribell^®^ also provides immediate feedback on electrode contact quality, prompting the user to adjust as necessary.

POC-EEG systems are therefore more rapidly deployed without specialized technologists, making them practical in emergency, intensive care, and even pre-hospital settings, such as by emergency medical services personnel in the field. Reported setup times for current POC-EEG devices are generally within 2–10 min.

These advantages in speed and simplicity have translated into successful real-world applications across a variety of acute care settings. For example, the Prehospital Implementation of Rapid EEG (PHIRE) study evaluated the feasibility of Ceribell^®^ use in EMS for patients with suspected stroke, altered mental status, or seizure [16]. Clinicians were able to apply the 10-electrode device and begin recording within an average of 2.5 min, and 94% of recordings were interpretable. Additionally, 91% of recordings had at least one channel per hemisphere free of artifact for over one minute, and 76% were artifact-free for five minutes or more. EMS providers reported the device as easy to use [16]. Another publication demonstrated the effective use of a 2-channel POC-EEG in pediatric emergency care, with all patients (*n* = 36) having leads placed in under 3 min and 77.8% of clinicians rating the application process as “easy [17].”

Recent advancements have driven the evolution of POC-EEG from simplified bedside monitors to sophisticated, wearable, wireless systems with embedded analytics. These systems have also introduced integrated AI software that supports real-time waveform visualization, cloud-based remote review, and, in some cases, full montage systems and automated seizure burden tracking with real-time bedside notifications—features that empower non-neurologists to initiate care sooner [18].

A major innovation across newer platforms is the integration of automated interpretation algorithms. These algorithms use machine learning techniques to detect seizure burden trends. AI-assisted tools enhance interpretability and accessibility, enabling use by non-experts and supporting clinical decision-making in settings where neurologists may not be immediately available. However, there are some concerns. Current AI algorithms tend to perform well at the extremes of seizure burden (>90% or <10%), but are less reliable at detecting EEG abnormalities that are subtle or isolated [19]. Additionally, these systems may misinterpret physiological artifacts or non-seizure EEG patterns such as periodic discharges or triphasic waves, leading to false positives and potentially unnecessary treatment. Therefore, these systems should augment and not replace clinical interpretation and should be applied within the technical constraints of the underlying montage and the patient’s clinical context [20,21].

EEG sonification is an additional innovation designed to assist non-specialists in interpretation. In a prospective clinical trial, ICU staff using sonification feature were able to rapidly and accurately detect seizures, with sensitivity and specificity comparable to expert EEG readers [22]. Notably, even clinicians without formal EEG training could identify ongoing seizure activity by listening to the sonified EEG, supporting the utility of this approach for rapid bedside evaluation [22]. However, sonification is less effective in distinguishing seizure-like patterns such as periodic discharges or artifacts [21].

Diagnostic variability remains a challenge with POC-EEG [21]. Reduced montage systems, while improving feasibility, inherently limit spatial resolution, potentially missing focal abnormalities [23]. As of December 2025, ZetoEEG^®^ is the only FDA-approved POC-EEG system that offers a full montage with 21 electrodes. Additionally, signal degradation and artifact from patient movement and electrical interference remain significant challenges for POC-EEG, with a proportion of recordings being classified as uninterpretable due to these issues [21]. While dry electrodes offer convenience, they may contribute to high impedance, increasing susceptibility to noise and motion-related artifacts [24]. Most platforms utilize impedance monitoring to mitigate artifact, and some platforms utilize built-in AI-based software for artifact detection and removal.

### 3.1. Comparison Between Conventional EEG and POC-EEG

**Figure 1:**
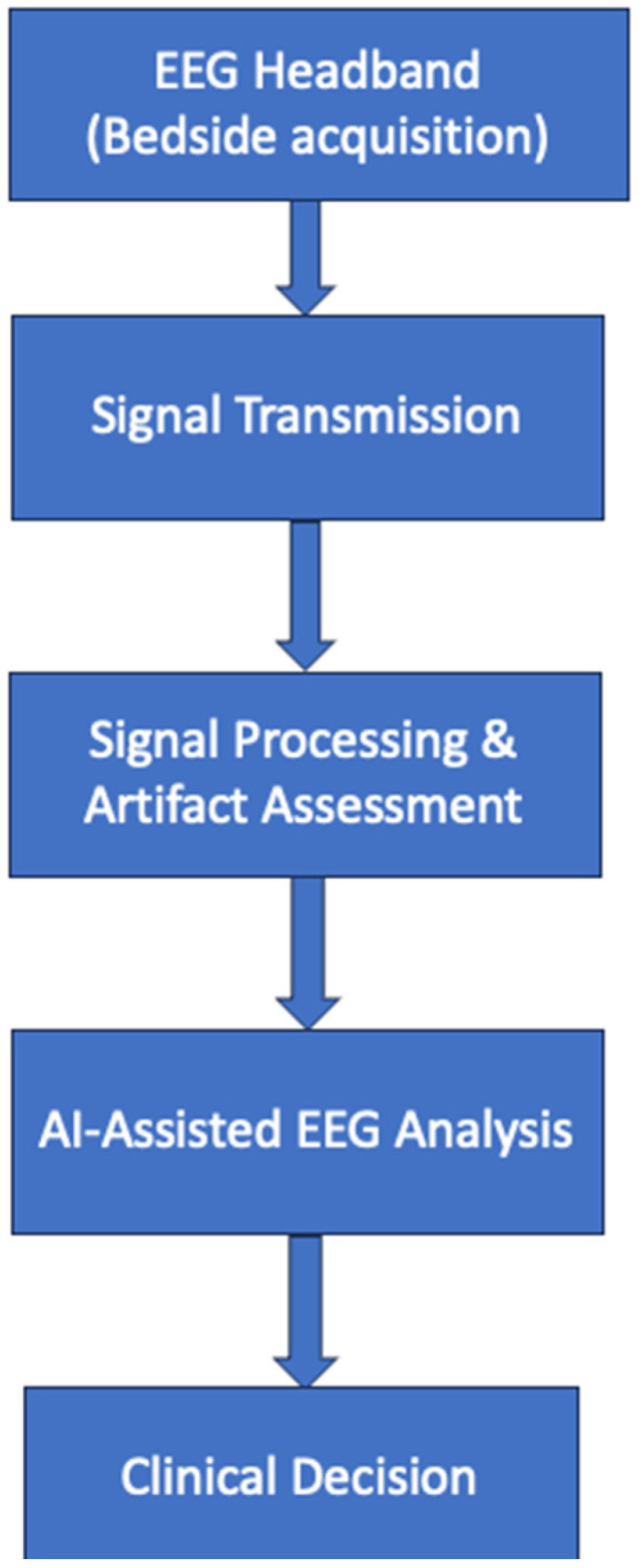
Schematic illustrating the conceptual workflow of point-of-care EEG acquisition and AI-assisted clinical decision support. EEG signals are acquired at the bedside, processed for signal quality, analyzed using automated methods, and reviewed by clinicians in the context of the patient’s clinical presentation. This schematic is illustrative only and does not represent any specific commercial system.

Conventional EEG and POC-EEG differ in several key aspects of patient care, including setup time, cost, accessibility, patient comfort, and the range of monitoring environments. Many new mobile EEG solutions require significantly less setup time. Figure 1 exemplifies a general POC-EEG setup. For example, compared to the standard scalp EEG setup, the Rapid EEG CeribAir^®^ headset reduced setup time by approximately 303 min. Overall, mobile EEG systems are associated with lower costs, which is particularly valuable for patient care in populations with limited access to conventional EEGs. Features such as wireless connectivity in mobile EEG devices contribute to a positive patient experience and increased comfort during monitoring. Additionally, these devices can be used in various settings, including the ICU, ED, and even at home, highlighting their easy-to-use design [10].

POC-EEG has been shown to provide diagnostic quality equivalent to conventional EEG. A prospective observational study found that POC-EEG and conventional systems were virtually equivalent in time-aligned recordings. Quantitative metrics such as Hjorth parameters, spike count, baseline wander, and kurtosis were statistically similar. Additionally, POC-EEG demonstrated significantly less 60 Hz electrical noise than conventional systems in both laboratory and ICU settings [25]. The DECIDE trial examined the use of POC-EEG in critically ill patients with NCS. The study found that in cases where seizures were detected by both types of EEG, the recordings were visually equivalent for diagnostic purposes. Discrepancies in seizure detection were primarily attributed to clinical factors, such as medications and timing, rather than limitations of the device itself [14].

A study comparing POC-EEG to the standard 21-electrode EEG using the 2HELPS2B score (for seizure risk prediction) found that POC-EEG performed similarly well in predicting seizures, indicating its potential value in rapid triage. However, due to fewer electrodes, the POC-EEG may miss focal abnormalities originating from or spreading to central and parietal regions. Based on this study, POC-EEG could be effective for urgent decision-making but should not be considered a substitute for a standard comprehensive EEG [26].

In another study, neurology residents with no prior POC-EEG experience initially assessed whether a group of 164 ICU patients were having seizures based on clinical signs alone. The residents then used POC-EEG to check their accuracy. After using POC-EEG, residents improved their seizure detection sensitivity from 78% to 100%. Furthermore, the POC-EEG results were determined within minutes, compared to the hours required by traditional EEG methods. However, the study notes that POC-EEG uses fewer electrodes than traditional EEG, which means it could miss some localized seizures [27].

A POC-EEG system was also implemented in a community hospital to assess potential clinical and financial benefits. In such settings, limited EEG technology often leads to patient transfers due to concerns for seizures, increasing both costs and morbidity. This study demonstrated that a POC-EEG system led to an absolute decrease in transfers to tertiary hospitals. This reduction in transfers saved money and could potentially offset the cost of implementing a POC-EEG system in community hospitals. Furthermore, increased detection of seizures allowed patients to receive more immediate and appropriate treatment, leading to overall better patient outcomes [13].

### 3.2. Artificial Intelligence-Assisted Interpretation of POC-EEG

#### 3.2.1. Rationale and Scope of AI in POC-EEG

Artificial intelligence (AI) tools have been increasingly incorporated into point-of-care EEG (POC-EEG) systems to address delays in EEG interpretation and limited access to expert electroencephalographers in emergency and critical-care settings. In patients with altered mental status, suspected non-convulsive seizures, or non-convulsive status epilepticus (NCSE), timely recognition of ongoing ictal activity is critical, yet conventional EEG interpretation may be delayed by hours.

AI-assisted POC-EEG tools are therefore designed to support rapid bedside decision-making, particularly during off-hours or in resource-limited environments. Importantly, these tools are intended to augment clinical assessment rather than replace expert EEG interpretation, and their outputs must be interpreted in the context of electrode montage, signal quality, and the patient’s overall clinical presentation. To improve clarity and consistency, AI tools discussed below are described using a uniform framework: algorithmic concept, clinical output, and key limitations [28].

#### 3.2.2. AI-Assisted Seizure-Burden Detection on Reduced-Montage POC-EEG: Clarity AI (Ceribell^®^)

The Clarity AI algorithm (Ceribell^®^) quantifies seizure activity by calculating the “seizure burden,” defined as the proportion of 10 s EEG epochs containing ictal activity across all channels within the preceding five minutes. An alert is generated when this seizure burden exceeds 90%, corresponding to at least 4.5 min of seizure activity within the 5 min window, which is indicative of impending status epilepticus rather than isolated or brief seizures. This cumulative seizure burden metric reflects sustained epileptiform activity, which correlates more strongly with neuronal injury and clinical urgency than transient discharges [25,28,29].

Clarity AI is explicitly designed to support rapid bedside decision-making in acute neurological care and is not intended for the detection of isolated epileptiform discharges or brief seizure episodes [29]. The algorithm’s training dataset comprises thousands of hours of expert-annotated EEG recordings, enabling robust detection of high seizure burdens while minimizing false positives from physiological artifacts or non-ictal rhythmic patterns [20,25]. A high seizure burden represents sustained electrographic ictal activity associated with increased neurological risk under ACNS criteria, but should be distinguished from ILAE conceptual definitions of status epilepticus, which are based on time-dependent pathophysiology rather than EEG burden alone (see Table 1) [25,27,28].

Version 7.0 of Clarity AI, released in December 2024, incorporated algorithmic enhancements that improved sensitivity for detecting impending status epilepticus, particularly in challenging clinical populations such as post-cardiac arrest patients. The system has received FDA clearance for seizure detection in patients aged one year and older, based on validation in a large pediatric cohort exceeding 1700 cases, making it the first POC-EEG AI system cleared across the full pediatric and adult age spectrum. In November 2025, FDA clearance was further expanded to include pre-term neonates, supported by EEG data from over 700 neonates, representing the largest neonatal seizure-detection dataset used for regulatory approval to date. (https://ceribell.gcs-web.com/news-releases/news-release-details/fda-clears-ceribells-claritytm-algorithm-pediatric-patients, accessed 30 September 2025).

Clinical validation studies demonstrate high diagnostic accuracy of Clarity AI. In a multicenter retrospective analysis of 1340 adult Ceribell^®^ recordings, the algorithm achieved a sensitivity of 96.0% and specificity of 94.8% for status epilepticus detection, with a negative predictive value of 99.9% [14,20]. Another retrospective cohort of 1148 adult EEGs reported Clarity alerts in 19 of 21 status epilepticus cases and correctly ruled out seizures in 726 of 727 non-status epilepticus recordings, underscoring its clinical reliability [14].

Ceribell^®^ is one example of a rapidly deployable POC-EEG system; other platforms use different electrode configurations, signal processing approaches, and analytic frameworks to address similar clinical needs.

#### 3.2.3. AI-Assisted Seizure Detection on Higher-Channel POC-EEG: Zeto One^®^ (NeuroPulse™)

Zeto One^®^ is a full 10–20 EEG system designed to increase electrode density, particularly over parasagittal and central regions, thereby addressing some of the spatial limitations inherent to reduced-montage EEG systems. The device is FDA-cleared and incorporates Encevis technology, developed by the Austrian Institute of Technology. The NeuroPulse™ AI software embedded in Zeto One^®^ was trained on over 21,400 h of EEG data from more than 800 patients and validated on an independent cohort of 81 patients, demonstrating robust seizure-detection performance in acute-care settings (https://www.prnewswire.com/news-releases/zeto-unveils-neuropulse-the-first-ai-powered-status-epilepticus-software-using-full-10-20-eeg-in-collaboration-with-encevis-302417953.html, accessed on 30 September 2025).

Unlike gel-based systems such as Ceribell^®^, Zeto One^®^ uses dry electrodes, facilitating rapid application and patient comfort. It is engineered for critical and ED environments, supporting long-duration EEG monitoring, real-time AI-driven seizure alerts, and cloud integration with synchronized video and audio capture for remote expert review (https://www.neurologylive.com/view/fda-clears-zeto-adjustable-one-headset-eeg-brain-monitoring, accessed on 30 September 2025). In contrast, the Zeto WR19 system is optimized for short, routine EEG examinations in outpatient or controlled clinical settings, while maintaining wireless functionality and dry electrodes for ease of use. (https://zeto-inc.com/device/, accessed on 30 September 2025).

Technical validation of the Zeto platform has been reported by Nadasdy et al. [24] who compared Zeto WR19 (zEEG) with conventional EEG (cEEG) in 15 patients during simultaneous 30 min recordings. Signal-quality metrics included Hjorth parameters, waveform correlation, spectral density, signal stability, and signal-to-noise ratio (SNR). The zEEG system demonstrated non-inferior signal quality relative to cEEG, with significantly reduced sensitivity to 60 Hz electrical noise. Waveform correlation coefficients exceeded 0.6 (*p* < 0.001), spectral correlations were greater than 0.99 (*p* < 0.001), and the mean SNR was 4.82 dB higher in zEEG, representing a 16% improvement in signal quality. While these findings support the technical non-inferiority of zEEG relative to cEEG, the study’s small sample size and short recording duration limit generalizability.

The NeuroPulse™ AI algorithm embedded in Zeto One^®^ tracks seizure burden with high reported accuracy. Retrospective clinical data from 81 acute-care patients demonstrated a positive percent agreement of 82.6% and negative percent agreement of 91.4% relative to expert EEG annotations [24].

Compared with reduced-montage POC-EEG systems, the enhanced electrode coverage of Zeto One^®^ enables improved detection of focal epileptiform activity, while dry electrodes and cloud-based workflows facilitate rapid deployment and remote interpretation across diverse clinical environments.

ZetoEEG^®^ thus represents an alternative design philosophy emphasizing expanded electrode coverage and AI-assisted seizure detection; however, as with other POC-EEG platforms, no single system has been shown to be optimal across all clinical scenarios, and performance remains dependent on signal quality, artifact burden, workflow constraints, and availability of expert interpretation (https://aesnet.org/abstractslisting/automatic-detection-of-nonconvulsive-status-epilepticus-and-seizure-burden-monitoring-using-a-full-montage-eeg-system, accessed on 30 September 2025).

### 3.3. BrainScope

BrainScope^®^ is an FDA-cleared EEG-based medical device designed for the evaluation of mild traumatic brain injury (mTBI), including concussion and intracranial hemorrhage, through advanced artificial intelligence algorithms applied to brain electrical activity (BrainScope Science & Technology) (https://www.brainscope.com/sciencetechnology, accessed on 30 September 2025). The device uses an 8-electrode disposable headset positioned over frontal and fronto-temporal scalp regions, analyzed by proprietary classifiers: the Structural Injury Classifier (SIC) predicts CT-positive intracranial injury, and the Brain Function Index (BFI) quantifies functional brain impairment consistent with concussion [30].

A prospective validation study involving 720 adult patients presenting within 72 h of closed head injury (Glasgow Coma Scale 12–15) demonstrated that the SIC binary classifier achieved a sensitivity of 92.3% and a negative predictive value (NPV) of 96.0% for any CT-detectable intracranial injury. A ternary classifier showed even higher sensitivity (98.6%) and NPV (98.2%) for traumatic hematomas. The algorithm exhibited superior specificity compared to standard CT decision rules, supporting its utility for triage and clinical decision-making in emergency settings [30].

Hack et al. (2017) [31] reported that combining BrainScope output with clinical parameters such as loss of consciousness (LOC) improved prognostic accuracy for TBI by 83% compared to LOC alone. Given that over 90% of CT scans for mild head injury are negative, BrainScope offers a promising radiation-free alternative to reduce unnecessary imaging. Its portability and ease of use make it suitable for ED, urgent care, sports, and military settings [31].

Large-scale clinical studies funded by the Department of Defense and NFL/GE Head Health Challenge enrolled over 1700 patients aged 13–25, demonstrating BrainScope’s ability to objectively assess concussion severity and recovery trajectory using multimodal EEG biomarkers [31] (https://www.medical-xprt.com/news/brainscope-completes-large-scale-clinical-studies-of-concussion-in-universities-high-schools-and-con-1056469, accessed on 30 September 2025).

In 2025, BrainScope launched an advanced deep learning platform integrating large neural models to enhance EEG analysis across brain health conditions including concussion, stroke, and early Alzheimer’s disease. This platform aims to eliminate the need for specialized qEEG interpretation and expand clinical utility, although current FDA clearance remains focused on mTBI and concussion assessment [31] (https://secure.businesswire.com/news/home/20250611423351/en/BrainScope-Expands-Board-and-Launches-Advanced-Deep-Learning-Platform-to-Enhance-Brain-Health-Insights, accessed on 30 September 2025).

### 3.4. Reduced-Montage Wireless EEG Systems for Rapid Bedside Monitoring: VitalEEG™ and CerebAir (AE-120A)

Nihon Kohden’s CerebAir (AE-120A) EEG headset is an FDA-cleared Class II wireless system designed for continuous neurophysiologic monitoring in ICU settings via integration with designated EEG platforms (https://fda.innolitics.com/submissions/NE/subpart-b%E2%80%94neurological-diagnostic-devices/OMC/K183529, accessed on 30 September 2025). The system employs an adjustable 8-electrode configuration with pre-filled gel cups and Bluetooth transmission, allowing rapid application by non-specialist staff without extensive skin preparation. Real-time EEG display and electrode-status feedback enable remote interpretation and monitoring (https://www.medicalexpo.com/prod/nihon-kohden-europe/product-69520-853475.html, accessed on 30 September 2025).

VitalEEG™, introduced in the U.S. in September 2019, is an FDA-cleared wireless headset with up to eight electrodes featuring Bluetooth connectivity and remote access, enabling setup within five minutes. It is optimized for rapid neurological assessment in acutely unresponsive patients, supporting emergent clinical decision-making (https://us.nihonkohden.com/nkweb/en/USD/products/neurology/vitaleeg-amplifier-ae-120a, accessed on 30 September 2025).

Clinical feasibility and diagnostic performance of CerebAir swEEG have been evaluated in acute-care settings. Welte et al. [32] studied CerebAir application in 100 adult emergency-department patients presenting with seizures or altered consciousness, with EEG applied by non-specialist staff. Median application time was 7 min (range 4–20). In 55 paired recordings, swEEG findings matched conventional 21-electrode EEG in 87.3% of cases. In 9.3% of patients for whom standard EEG was delayed or unavailable, swEEG identified pathological patterns—including status epilepticus—that led to immediate treatment decisions [32].

A 2020 prospective ICU study compared CerebAir application by ICU physicians (n = 20) with technician-applied swEEG (n = 20). Nurse- or physician-applied CerebAir achieved a significantly shorter mean setup time (6.2 ± 1.1 min) compared with standard application (10.4 ± 2.3 min, *p* < 0.0001), with equivalent detection rates of EEG abnormalities (35% in both groups). Artifact corrections were more frequent in the CerebAir group, but monitoring remained feasible without technician support and with comparable overall signal interpretability [33].

The National Institute for Health and Care Excellence (NICE) supports CerebAir use for continuous EEG monitoring in ICU settings, citing faster application times and acceptable sensitivity and specificity across three observational studies involving 157 adults. Reported adverse effects were limited to minor, reversible skin irritation following prolonged use (>15 h). However, NICE also emphasizes that clinical outcome impact has not yet been established, and that device acquisition costs (approximately £20,000–£30,000) and ongoing consumable expenses represent important economic considerations (https://www.nice.org.uk/advice/mib279/resources/cerebair-for-continuous-eeg-monitoring-in-intensive-care-pdf-2285965822743493, accessed on 30 September 2025).

VitalEEG™ and CerebAir exemplify reduced-montage POC-EEG systems optimized for speed, accessibility, and deployment by non-specialist staff. The primary strength of these systems lies in rapid bedside initiation, which can shorten time to EEG assessment and facilitate early clinical decision-making in critically ill or unresponsive patients.

However, the limited number of electrodes constrains spatial resolution and increases the risk of missed focal, parasagittal, or deep-seated epileptiform activity, particularly when compared with higher-channel or full 10–20 EEG systems. Reduced-montage recordings may also be more susceptible to artifacts, requiring increased correction and careful interpretation, as reflected by higher artifact-adjustment rates in ICU studies [32,34]. While signal quality has generally been sufficient for clinical screening and monitoring, noise-to-signal ratio and artifact burden remain important considerations, especially during prolonged monitoring or in agitated patients.

As with other POC-EEG platforms, VitalEEG™ and CerebAir should therefore be viewed as adjunctive tools that enhance access to EEG in time-sensitive settings rather than as replacements for comprehensive conventional EEG interpreted by trained neurophysiologists.

### 3.5. BrainWatch, Natus

Natus BrainWatch™, a POC-EEG decision support system, gained FDA clearance, for rapid detection of electrographic seizures and status epilepticus (https://practicalneurology.com/news/the-fda-clears-an-eeg-decision-support-tool-that-identifies-seizure-and-status-epilepticus/2470661/, accessed on 30 September 2025). The system features a wireless, wearable EEG headset that integrates seamlessly with the Natus NeuroWorks^®^ platform, which is widely deployed across large health systems and community practices. This integration enables wireless setup in under five minutes, facilitating early EEG acquisition in time-critical environments (https://www.prnewswire.com/news-releases/natus-to-debut-brainwatch-point-of-care-eeg-solution-at-aes-302322548.html, accessed on 30 September 2025). BrainWatch incorporates FDA-cleared Persyst 15 AI algorithms for on-device seizure detection, seizure-burden estimation, and automated recognition of electrographic status epilepticus, providing near-real-time alerts to bedside clinicians and enabling remote neurologist review via NeuroWorks (https://practicalneurology.com/news/the-fda-clears-an-eeg-decision-support-tool-that-identifies-seizure-and-status-epilepticus/2470661/, accessed on 30 September 2025).

Early implementations demonstrated full referential EEG analysis compliant with ACNS guidelines and support for post-recording remontaging, which improves localization and artifact identification during expert review (https://www.massdevice.com/natus-neuro-launches-brainwatch-ai-eeg/, accessed on 30 September 2025). Operational observations from rural hospitals reported fewer inter-facility transfers and increased confidence in ruling out seizures when BrainWatch readings were negative, though formal clinical outcomes studies are pending (https://xtalks.com/fda-clears-natus-brainwatch-for-bedside-detection-of-electrographic-status-epilepticus-4541/, accessed on 30 September 2025).

BrainWatch is optimized for rapid deployment and decision support, aligning with best-practice recommendations for early seizure identification in critically ill patients, among whom subclinical seizures occur in up to 30%, and responsiveness to first-line treatment declines sharply with delay (https://natus.com/neuro/brainwatch/, accessed on 30 September 2025). However, as a POC-EEG system, its performance remains influenced by electrode density, artifact burden, and signal quality, particularly when used by non-specialist staff.

Compared with full conventional EEG, POC-EEG systems such as BrainWatch may face limitations in spatial resolution and increased susceptibility to movement-related and muscle artifacts, which can affect noise-to-signal ratio and necessitate expert review for definitive interpretation. Accordingly, BrainWatch should be viewed as a triage and decision-support tool that expands access to EEG in underserved settings rather than as a replacement for comprehensive EEG interpreted by trained neurophysiologists.

### 3.6. Multi-Channel Wireless EEG Platforms and AI Readiness: Enobio Dx (Neuroelectrics)

The Enobio Dx (Neuroelectrics, Barcelona, Spain) is an FDA-cleared, wireless, battery-powered EEG system designed for clinical neurophysiological monitoring in both hospital and ambulatory settings (https://www.accessdata.fda.gov/cdrh_docs/pdf16/K162681.pdf, accessed on 30 September 2025). The platform supports 8, 20, or 32 channels, with a DC-coupled bandwidth of 0–125 Hz, a sampling rate of 500 samples per second, and 24-bit analog-to-digital conversion, yielding a dynamic range of 0.05 μV and measurement noise below 1 μV RMS (https://www.vistamedical.at/upload/enobio-technical-data.pdf, accessed on 30 September 2025).

High input impedance (1 GΩ) and a common-mode rejection ratio of −115 dB support detection of low-amplitude and high-frequency neural signals. EEG digitization occurs at the scalp interface, reducing analog transmission artifacts, with data transmitted via Wi-Fi (IEEE 802.11g) or USB for real-time analysis. Onboard microSD storage enables extended “Holter-mode” recordings up to 20 h, supporting long-duration monitoring in real-world environments (https://www.vistamedical.at/upload/enobio-technical-data.pdf, accessed on 30 September 2025).

Electrode options include dry, semi-dry, and gel-based configurations, with ergonomic caps available in six sizes and 10-10 montage layouts. Setup times are minimized: dry electrodes can be applied in 1–4 min, semi-dry in 3–5 min, and gel-based in 8–25 min, depending on channel count [34]. The system integrates a 3-axis accelerometer (100 S/s) and is compatible with tDCS/TMS, real-time ERP mapping, and third-party platforms (OpenVibe, BCI2000, EEGLAB, NEPy) via Lab Streaming Layer or TCP/IP (https://www.vistamedical.at/upload/enobio-technical-data.pdf, accessed on 30 September 2025).

A prospective observational study (NCT03038191) at Tel Aviv Sourasky Medical Center evaluated the feasibility of Enobio 8^®^ for continuous EEG and physiological monitoring in adults with epilepsy. Twenty-eight patients (18–80 years) expected to experience seizures underwent continuous EEG for up to 120 h in a video-EEG unit and up to 336 h at home. The primary endpoints were the proportion of artifact-free EEG epochs and the integrity of physiological data streams. Although the trial was terminated early due to funding cessation, interim analysis demonstrated high-fidelity EEG acquisition and minimal signal dropout in both hospital and ambulatory settings, supporting the technical feasibility of extended-duration, real-world EEG monitoring and providing a foundation for future algorithmic seizure detection research.

While Enobio’s higher channel counts offer improved spatial resolution compared with reduced-montage POC-EEG systems, increased electrode density introduces greater susceptibility to motion artifacts, electrode impedance variability, and environmental noise, which can affect noise-to-signal ratio and increase the burden of artifact rejection. Longer setup times for higher-channel configurations may also limit feasibility in time-critical ED scenarios.

Accordingly, Enobio is best positioned as a research-ready and hybrid clinical platform capable of supporting advanced EEG analytics and future AI development, rather than as a rapid-deployment seizure-triage device. As with other high-channel EEG systems, optimal clinical performance depends on workflow integration, artifact management, and expert interpretation.

### 3.7. Consumer-Grade Wearable EEG: Emotiv MN8

The Emotiv MN8 is a consumer-grade, non-medical wearable EEG device designed primarily for cognitive research, neurofeedback, and personal wellness applications rather than clinical diagnostics or therapeutic use. It employs two dry, non-toxic conductive elastomer sensors positioned bilaterally in the external auditory canals, enabling discreet brainwave acquisition during daily activities (https://www.emotiv.com/pages/quick-start-guide-mn8?srsltid=AfmBOoqk4wsZKPU_ZF5znEO0TNz0TJbANC2rw395vDD7UW_H3PhbDTbh, accessed on 30 September 2025). The device samples EEG signals internally at 8 kHz, downsampled to 128 Hz, with 14-bit resolution and a least significant bit sensitivity of approximately 0.51 µV, covering a frequency range of 0.16 to 45 Hz and incorporating a six-axis motion sensor to detect and mitigate movement artifacts [35]. Connectivity is via Bluetooth Low Energy, supporting up to 6 h of continuous operation on a rechargeable battery (https://www.emotiv.com/pages/quick-start-guide-mn8?srsltid=AfmBOoqk4wsZKPU_ZF5znEO0TNz0TJbANC2rw395vDD7UW_H3PhbDTbh, accessed on 30 September 2025). Although the MN8 holds CE marking for compliance with EU safety standards, this certification pertains only to consumer safety and does not imply clinical validation or FDA clearance for medical use; the manufacturer explicitly states it is not intended for diagnosis, monitoring, or treatment of medical conditions [31,36]. While widely used in cognitive wellness and research settings, peer-reviewed clinical validation studies assessing its signal quality and diagnostic utility compared to clinical EEG systems remain limited, and its two-channel configuration inherently restricts spatial resolution and clinical applicability [37,38].

From a clinical perspective, the MN8’s two-channel configuration severely limits spatial resolution, rendering it unsuitable for detection of focal epileptiform activity, seizure localization, or comprehensive neurophysiological assessment. While widely used in cognitive and wellness research, peer-reviewed clinical validation studies comparing MN8 signal quality to medical-grade EEG systems remain limited. As such, the MN8 should be regarded as a research and wellness tool rather than a POC-EEG system applicable to acute neurological care [37,38].

### 3.8. Open-Source Research EEG Platforms: Ultracortex Mark IV (OpenBCI)

The OpenBCI Ultracortex Mark IV is an open-source, 3D-printable EEG headset designed for research and engineering development, not for clinical or diagnostic use. It is explicitly not FDA-cleared and is marketed as a customizable platform compatible with OpenBCI biosensing boards (Cyton or Cyton+Daisy) supporting up to 16 EEG channels placed according to the international 10–20 system. The headset uses dry electrodes with adjustable mounts for rapid setup and is widely used in neuroscience research and brain–computer interface applications.

Despite its research-grade capabilities and use in peer-reviewed studies, it lacks regulatory clearance for medical use and is not considered a finished consumer or medical product [39]. While the Ultracortex enables flexible electrode configurations and supports higher channel counts than many consumer devices, its signal quality, electrode stability, and artifact control depend heavily on user expertise and hardware configuration. Variability in electrode contact, motion artifacts, and environmental noise can significantly affect noise-to-signal ratio, limiting reliability in uncontrolled or clinical environments. Accordingly, the Ultracortex Mark IV should be considered a research-grade experimental platform, not a POC-EEG solution for clinical deployment.

### 3.9. Portable Clinical EEG Systems: WAVi Scan

The WAVi SCAN is a portable, multi-channel EEG system with FDA clearance for point-of-care use in both clinical and non-clinical environments. It supports up to 19 EEG channels using standard 10–20 electrode placement and enables rapid acquisition of resting-state EEG and event-related potentials (ERPs), including auditory evoked potentials. The system integrates hardware and software for real-time signal acquisition, artifact rejection, and basic signal processing to facilitate efficient neurophysiological assessment [40].

WAVi SCAN occupies an intermediate position between reduced-montage POC-EEG systems and full conventional EEG. While higher channel counts improve spatial resolution compared with rapid-deployment headsets, the system remains constrained by short recording durations, simplified analytic outputs, and limited applicability for continuous ICU monitoring or seizure burden assessment. Its role is best suited to screening, baseline assessment, and neurocognitive evaluation, rather than acute seizure triage or status epilepticus management.

### 3.10. Wearable Brain Sensing Devices

POC-EEG systems such as Ceribell^®^ are FDA-approved devices used clinically in ICU and emergency settings, prioritizing rapid setup (typically 8–10 electrodes), early seizure detection, and expedited clinical decision-making. These systems often incorporate AI algorithms for seizure detection and are generally covered by health insurance [37,38]. In contrast, consumer-oriented wearable EEG devices (e.g., NeuroSky MindWave, Muse S Gen 2, Emotiv Insight, OpenBCI Galea) target wellness, research, and brain–computer interface applications. These devices typically employ dry or semi-dry electrodes with 1–16 channels, lack FDA regulation, and are not reimbursed by insurance, with prices ranging from hundreds to thousands of dollars [40]. Research-grade wearable systems like Kernel Flow and Neuroelectrics Enobio integrate advanced sensing modalities such as wireless EEG and functional near-infrared spectroscopy (fNIRS) for neuroscience research [28,40].

Despite increasing popularity, wearable EEG devices face significant limitations that constrain clinical applicability. Reduced channel counts compromise spatial resolution and limit detection of subtle or focal brain activity. Dry electrode technology, while improving usability, is more susceptible to motion artifacts and impedance variability, affecting noise-to-signal ratio, particularly during prolonged or real-world use. Limited battery life further restricts continuous monitoring, and the absence of standardized data formats hampers cross-platform integration and comparability.

The absence of standardized data protocols further hampers data comparability and integration. Additionally, limited battery life restricts prolonged real-world use. As wearable EEG adoption grows, privacy and data security concerns become increasingly salient due to the collection and transmission of sensitive neurophysiological data. Establishing standardized acquisition protocols, validation benchmarks, and ethical safeguards will be essential for responsible deployment and for delineating the boundaries between wellness, research, and clinical neurophysiology applications [40].

## 4. Demographics of POC-EEG Use

POC-EEG is increasingly utilized across diverse patient populations and clinical settings, particularly where conventional EEG access is limited or delayed. Older adults constitute the majority of POC-EEG users in acute care, with common indications including non-convulsive seizures, status epilepticus, unexplained altered mental status, post-cardiac arrest evaluation, and acute stroke for differentiating seizures from stroke mimics [21]. A cohort study of 88 patients in two New Jersey community hospitals reported a mean age of 57 years, with 90% of recordings performed in intensive care units; rapid bedside EEG is critical in this group due to atypical presentations and baseline cognitive impairments that complicate seizure recognition [13]. Pediatric use of POC-EEG is rising, with retrospective analyses demonstrating feasibility and efficacy of reduced-lead EEG montages for detecting non-convulsive status epilepticus and other critical neurologic conditions such as hypoxic–ischemic encephalopathy and traumatic brain injury [17]. However, pediatric recordings exhibit higher artifact rates, primarily from motion and electrode contact challenges, warranting further comparative studies with conventional EEG [17]. Demographically, the New Jersey cohort included 55% male, 43% female, and 2% transgender or unspecified individuals, though racial and ethnic data were not reported—a common gap in neurodiagnostic literature [13]. Although racial and ethnic data were not reported, the lack of this kind of demographic reporting remains a known issue in neurodiagnostic literature [13]. Geographically, POC-EEG is often deployed in underserved, resource-limited hospitals such as those in Camden and Vineland, NJ, where socioeconomic diversity and high poverty rates prevail. In these settings, POC-EEG has reduced inter-hospital transfers and generated annual savings exceeding $37,000, underscoring both clinical and economic benefits [13].

## 5. Clinical Applications of POC-EEG

**Figure 2:**
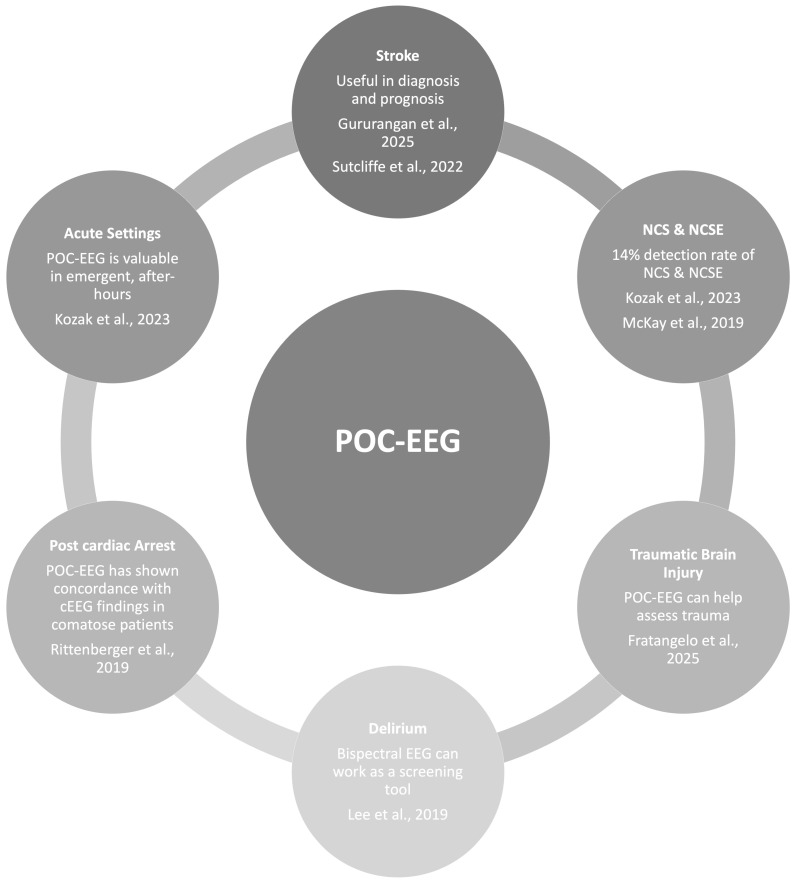
Clinical applications of POC-EEG in acute neurological care. POC-EEG enables rapid bedside assessment of cerebral electrical activity in a range of acute clinical scenarios, including NCS and NCSE, stroke, traumatic brain injury, delirium, post-cardiac arrest encephalopathy, and undifferentiated neurologic presentations in emergency or after-hours settings. The surrounding nodes summarize representative clinical contexts in which POC-EEG has demonstrated diagnostic or triage utility, with selected supporting studies shown for each application [8,21,41,42,43,44,45].

POC-EEG systems have demonstrated critical utility in emergency and acute care settings by enabling rapid identification of non-convulsive seizures and status epilepticus, thereby guiding timely antiseizure medication management (Figure 2). A retrospective cohort study involving 157 patients in an ED with limited access to EEG-trained neurologists found that POC-EEG detected seizures in 22 patients, including 10 cases of status epilepticus and 2 brief seizure events at monitoring initiation [8]. The presence of epileptiform activity on POC-EEG was significantly associated with escalation of antiseizure therapy (51.8%) compared to patients with normal or slow EEG activity (24.8%), while treatment de-escalation was more frequent in patients with normal or slow EEG (26.7%) than those with seizures (1.8%) [8,17]. These findings underscore the role of POC-EEG in refining clinical decision-making and optimizing pharmacologic interventions in real time.

For clarity, clinical urgency in this section is framed using ILAE conceptual time points, whereas EEG interpretation and device performance are discussed using ACNS operational criteria and seizure-burden metrics (see Table 1).

Further evidence from multicenter retrospective analyses comparing Ceribell^®^ POC-EEG to conventional EEG in critically ill ICU patients (N = 283) demonstrated a reduction in median ICU length of stay from 8.0 to 3.9 days (*p* = 0.003), improved functional outcomes with an 18% increase in favorable modified Rankin Scale scores, and a 19 h reduction in time to EEG acquisition [46]. Additionally, implementation of POC-EEG in community hospitals reduced emergent transfers for continuous EEG monitoring by approximately one patient per month, resulting in annual cost savings exceeding $37,000 [13,46].

In pediatric emergency care, systematic reviews and quality improvement studies confirm the feasibility and clinical utility of reduced-lead POC-EEG for detecting non-convulsive status epilepticus, hypoxic–ischemic encephalopathy, and metabolic encephalopathies, despite higher artifact rates primarily due to motion and electrode contact challenges [21,47]. A recent pediatric study reported 68% concordance between simplified two-channel POC-EEG and conventional EEG performed within 48 h, with seizure detection in 16% of cases and clinical management influenced in 60% [47].

### 5.1. NCS and NCSE

POC-EEG has been increasingly evaluated for the detection of NCS and NCSE in acute care settings. Its principal advantage lies in the rapid identification of seizure activity in patients with altered mental status, where clinical signs are frequently absent or nonspecific.

From an EEG standpoint, the ACNS defines electrographic seizures and electrographic status epilepticus using operational criteria based on seizure duration and seizure burden rather than elapsed clinical time. In this framework, seizure burden—typically expressed as the proportion of time occupied by ictal activity over a defined recording window—serves as a pragmatic marker of ongoing epileptic activity and neurological risk. This EEG-based approach is conceptually distinct from ILAE time points (t1 and t2), which are not intended as rigid diagnostic thresholds but as pathophysiological markers that vary by seizure type and semiology. Throughout this review, POC-EEG performance and AI-assisted detection are discussed primarily in relation to EEG burden-based criteria, rather than as replacements for ILAE conceptual definitions.

A retrospective study evaluating POC-EEG use in the ED reported a 14% detection rate of NCS among patients presenting with acute neurological deficits. Importantly, many of these seizures would likely have remained undiagnosed without EEG evaluation, underscoring the diagnostic value of POC-EEG acquisition in clinically ambiguous presentations [8]. These findings support early EEG deployment in patients with unexplained encephalopathy, where ongoing seizure activity may represent a reversible cause of neurologic dysfunction.

Similarly, a study employing a portable Brainmaster EEG system in the ED identified NCSE in 8% of evaluated patients, with EEG findings comparable in quality and interpretation to those obtained using conventional EEG systems [9]. This prevalence aligns with prior studies demonstrating that 8–48% of patients may continue to have nonconvulsive seizure activity following apparent termination of convulsive seizures [48,49,50]. Rapid bedside EEG thus plays a critical role in expediting the diagnosis of NCSE, enabling earlier antiseizure treatment and potentially mitigating secondary neuronal injury.

From a conceptual standpoint, POC-EEG is particularly well suited to patients who fit recently described high-risk frameworks for status epilepticus characterized by delayed diagnosis rather than early treatment failure. In Stage 1 Plus-type scenarios, patients may have ongoing seizures but present late in the disease course and have not yet received antiseizure therapy, a situation commonly encountered in suspected NCSE. In this context, POC-EEG triage serves two complementary roles: confirming persistent ictal activity early enough to support timely escalation of therapy in accordance with established guidelines, and excluding seizures in patients with altered mental status from other causes, thereby avoiding unnecessary exposure to sedatives or antiseizure medications. This dual function highlights POC-EEG as a pragmatic triage tool rather than a replacement for comprehensive EEG monitoring.

Further evidence supporting streamlined EEG approaches comes from a study comparing a rapid-application EEG electrode cap with standard EEG across the ED, inpatient wards, and intensive care unit (ICU). Both modalities identified NCSE in an equal number of patients; however, the median time to EEG acquisition was 86 min shorter with the cap-based system. This substantial reduction in setup time facilitated faster clinical decision-making and treatment initiation, without compromising diagnostic accuracy [41]. Time-to-diagnosis is particularly relevant in NCSE, where delays in EEG monitoring are independently associated with worse outcomes [51].

### 5.2. Stroke

POC-EEG may play an important role in the evaluation of patients presenting with acute neurological deficits, either as a complication of ischemic or hemorrhagic stroke or as a diagnostic tool for identifying stroke mimics, particularly NCS. In emergency care settings, conventional EEG acquisition is often limited by logistical constraints, including the need for specialized personnel and prolonged setup times, which may delay diagnosis and management. In this context, POC-EEG offers a practical adjunct to acute stroke evaluation by enabling rapid bedside assessment of cerebral electrical activity during time-sensitive clinical decision-making [42].

A retrospective observational cohort study by Gururangan et al. examined the utility of POC-EEG during stroke code activation in 70 patients presenting with acute focal neurological deficits due to either confirmed ischemic stroke or stroke mimics. Seizures or highly epileptiform patterns were identified in 6 of 38 patients (15.8%) with confirmed stroke, including 2 patients diagnosed with electrographic status epilepticus. Among 32 patients ultimately classified as stroke mimics, epileptiform abnormalities were detected in 11 cases (34.4%), including 2 patients with persistent expressive aphasia attributable to recurrent focal electroclinical seizures. These findings highlight the diagnostic value of POC-EEG in differentiating seizure-related phenomena from ischemic stroke during acute evaluation [42].

Epileptic seizures accompanied by postictal negative symptoms—such as aphasia, hemiparesis, or altered consciousness—represent one of the most common stroke mimics, accounting for approximately 20% of suspected stroke presentations. Accordingly, seizures should be consistently considered in the differential diagnosis of patients presenting with sudden neurological deficits, particularly in the absence of witnessed convulsive activity [52,53]. Misclassification of seizure-related deficits as stroke may lead to inappropriate management, while delayed recognition of seizures may postpone necessary antiseizure treatment.

Beyond seizure detection, surface EEG techniques, including POC-EEG, have demonstrated potential utility in acute stroke diagnosis and prognostication by identifying ischemia-related EEG changes and secondary epileptiform activity. However, despite encouraging results, robust clinical recommendations for routine EEG use in acute stroke pathways have not yet been established. Existing studies are limited by small sample sizes, heterogeneous patient populations, and variability in EEG technology and acquisition protocols [43].

### 5.3. TBI

POC-EEG has emerged as a scalable neurophysiological tool for the evaluation and prognostication of TBI, with applications spanning concussion assessment, early triage, and outcome prediction. EEG measures provide objective markers of cortical dysfunction associated with axonal injury and metabolic disruption, and may reduce reliance on computed tomography (CT) in selected patients by identifying those at low risk for structural lesions, thereby supporting more efficient imaging utilization [21].

In patients with moderate-to-severe TBI, EEG has demonstrated value in identifying covert residual cortical processing despite apparent clinical unresponsiveness. In a prospective observational cohort study by Claassen et al., EEG responses to spoken motor commands were detected in 16 of 104 (15%) comatose patients with acute brain injury in the intensive care unit (ICU). At 12-month follow-up, 44% of patients with EEG evidence of brain activation achieved functional independence (Glasgow Outcome Scale–Extended [GOSE] ≥ 4), compared with 14% of patients without detectable activation. These findings indicate that EEG-based detection of cognitive–motor dissociation provides prognostically meaningful information beyond standard bedside examination [54]. Although functional MRI may offer higher sensitivity for detecting covert consciousness, EEG is considerably more feasible in critically ill patients due to its portability, lower cost, and ease of repeated bedside application [54,55].

The integration of electrophysiological biomarkers into standard clinical assessment significantly improves prognostic discrimination in TBI. Prior studies have shown that EEG-derived metrics enhance outcome prediction compared with clinical indicators such as loss of consciousness and post-traumatic amnesia alone [31,56]. In a large prospective cohort of patients with moderate-to-severe TBI, early continuous EEG monitoring during ICU admission independently predicted poor long-term neurological outcomes with fair-to-good sensitivity and specificity, particularly when combined with established clinical, radiological, and laboratory variables [57]. These findings support EEG as a complementary modality for early risk stratification and individualized prognostication.

Recent advances in POC-EEG systems further expand the potential clinical impact of neurophysiological monitoring across the continuum of TBI care. In a prospective mixed-methods feasibility study involving 34 prehospital encounters, POC-EEG was applied by emergency medical services (EMS) personnel in a median time of approximately 2.5 min, with 94% of recordings deemed of sufficient quality for clinical interpretation. EEG findings influenced on-scene management and destination decisions, demonstrating the feasibility of neurophysiological assessment prior to hospital arrival in patients with acute neurological symptoms, including suspected [16].

### 5.4. Delirium

Delirium represents an acute disorder of cerebral function characterized by disturbances in attention and cognition, and remains highly prevalent in critical care environments. Contemporary estimates indicate that approximately 31% of intensive care unit (ICU) patients—and up to 80% of mechanically ventilated patients—experience delirium, which is independently associated with increased mortality, prolonged hospitalization, long-term cognitive impairment, and functional decline [58,59]. Despite this burden, delirium remains under-recognized, particularly in hypoactive phenotypes, due to the episodic nature of bedside assessments and the limitations of existing clinical screening tools, including reduced patient arousal, communication barriers, and confounding baseline cognitive impairment [60,61].

Point-of-care EEG (POC-EEG) offers an objective neurophysiological approach that addresses these limitations by enabling rapid, bedside assessment of global cortical dysfunction without reliance on subjective patient participation. Prior investigations employing simplified bispectral EEG (BSEEG), derived from two-channel recordings, have demonstrated that increased EEG slowing reliably distinguishes delirium from non-delirium states. In a prospective pilot study conducted in the ED, BSEEG achieved an area under the receiver operating characteristic curve (AUC) of 0.91, with sensitivity of 88.9% and specificity of 92.3% at an optimized threshold, when compared with standardized clinical and cognitive assessments [62]. Although constrained by small sample size and single-center design, these findings provided early evidence supporting the diagnostic utility of low-density EEG for delirium detection.

Subsequent studies expanded these observations by demonstrating prognostic significance. In a prospective cohort evaluation, elevated BSEEG scores independently predicted in-hospital mortality beyond clinical delirium diagnosis alone, suggesting that electrophysiological measures capture underlying cerebral dysfunction not fully reflected by symptom-based assessments [63]. Larger validation cohorts later confirmed that BSEEG positivity is associated with significantly increased 90-day and 1-year mortality, independent of delirium motor subtype and baseline comorbidity burden [64].

Importantly, newly published data further strengthen the clinical relevance of POC-EEG in delirium assessment. A prospective pilot study of a single-channel POC-EEG device in hospitalized older adults demonstrated significantly higher BSEEG scores among patients meeting delirium criteria using the 3-Minute Diagnostic Interview for Confusion Assessment Method (3D-CAM), with EEG-based screening outperforming routine bedside delirium tools in sensitivity [65]. In parallel, advances in automated EEG interpretation and continuous monitoring culminated in the first U.S. Food and Drug Administration (FDA) 510(k) clearance of an AI-powered, bedside EEG system for continuous delirium monitoring in the ICU. This system was validated in prospective cohorts exceeding 200 critically ill adults and enables real-time detection of EEG patterns associated with delirium, including hypoactive subtypes that are frequently missed by intermittent clinical screening (https://investors.ceribell.com/news-releases/news-release-details/ceribell-receives-fda-510k-clearance-first-its-kind-delirium, accessed on 8 January 2026).

### 5.5. Post-Cardiac Arrest

Cardiac arrest results in abrupt cessation of cerebral perfusion and carries a high risk of death and severe neurological injury. Survival following out-of-hospital cardiac arrest remains low, with reported rates below 10% despite advances in resuscitation and post-resuscitation care [66]. Among survivors, hypoxic–ischemic encephalopathy (HIE) represents the principal determinant of neurological morbidity and mortality and is frequently complicated by myoclonus, epileptic seizures, and status epilepticus [44,66,67].

A substantial proportion of post-cardiac arrest patients remain comatose during the early post-resuscitation period, rendering many seizures electrographic and clinically occult. Continuous EEG monitoring is therefore considered essential for timely seizure detection, assessment of background reactivity, and guidance of antiseizure and sedative therapy in this population [44,66,67]. In addition to its diagnostic role, EEG provides prognostically relevant information. Preserved or continuous background activity and EEG reactivity are associated with a higher likelihood of neurological recovery, whereas generalized suppression, periodic discharges, and burst-suppression patterns—particularly with identical bursts—are consistently correlated with unfavorable outcomes [44,68]. To standardize EEG-based prognostication, the American Clinical Neurophysiology Society (ACNS) has categorized post-cardiac arrest EEG patterns into benign, malignant, and highly malignant groups, facilitating structured risk stratification in comatose patients after return of spontaneous circulation [66,69].

Despite its clinical value, conventional intermittent or continuous EEG (cEEG) monitoring is resource-intensive, requires specialized personnel, and may be unavailable in many community or resource-limited hospitals, particularly outside regular working hours [44]. These limitations have prompted investigation into POC-EEG as a pragmatic alternative or adjunct for early neurophysiological assessment.

A prospective cohort study by Ward et al. evaluated the feasibility and cost-effectiveness of implementing a rapid-response POC-EEG system (Ceribell^®^) across two community hospitals. Among 88 patients requiring EEG monitoring when cEEG was unavailable—including individuals with post-cardiac arrest encephalopathy—21% demonstrated electrographic seizure activity on POC-EEG. Implementation of POC-EEG was associated with fewer interhospital transfers for EEG monitoring and an estimated annual cost savings of approximately $37,000, highlighting both its diagnostic and health-system value [13].

Complementary findings were reported by Rittenberger et al., who conducted a prospective comparison of POC-EEG and conventional cEEG in 95 comatose post-cardiac arrest patients [44]. Key background patterns, including continuous suppression and burst-suppression with identical bursts, were identified by both modalities. Electrographic seizures were detected in 2% of POC-EEG recordings and 4% of cEEG recordings, with fair inter-modality agreement (κ = 0.27). Notably, artifact limited interpretation in approximately 40% of POC-EEG studies, underscoring current technical constraints. These findings suggest that, while POC-EEG should not replace cEEG in centers where full monitoring is available, it can serve as a valuable adjunct for early neurophysiological assessment and triage in post-cardiac arrest patients [44].

### 5.6. Most Commonly Observed POC-EEG Abnormalities

POC-EEG is frequently deployed in acute and critical care settings to identify electrographic patterns indicative of seizure activity or global cerebral dysfunction. In comatose patients following cardiac arrest, several EEG abnormalities recur with notable consistency and carry important diagnostic and prognostic implications. In a cohort of 95 unresponsive post-cardiac arrest patients, the most prevalent EEG patterns included continuous background activity (21%), generalized suppression (14%), burst suppression (12%), and burst suppression with identical bursts (10%) [44,70].

Among these findings, burst suppression with identical bursts is regarded as particularly ominous. A multicenter study evaluating early EEG recordings in comatose post-cardiac arrest patients demonstrated that burst suppression with identical bursts—together with isoelectric and low-voltage EEG activity—predicted poor neurological outcome with 100% specificity and 100% positive predictive value [27,71]. In this cohort, all patients exhibiting these patterns failed to recover meaningful neurological function. Such prognostic accuracy is uncommon in clinical neurology and highlights the critical importance of early identification of highly malignant EEG phenotypes. Because these patterns are characterized by large-amplitude, bilaterally symmetric, and spatially widespread activity, they are typically well captured by reduced-channel POC-EEG systems despite limited electrode coverage [27,71].

POC-EEG is also effective in detecting electrographic seizures and periodic discharges, particularly in patients with unexplained encephalopathy or suspected nonconvulsive seizures. These abnormalities frequently involve broad cortical territories or occur at high temporal frequency, features that increase detectability using limited EEG montages. EEG patterns along the ictal–interictal continuum (IIC), including rhythmic or periodic discharges without clear evolution, are discussed separately from confirmed electrographic seizures and are not automatically classified as NCSE. Their interpretation requires clinical correlation, assessment of evolution, and response to treatment.

Comparative studies of reduced-montage versus conventional full-montage EEG have demonstrated high concordance for seizure identification when epileptiform activity is generalized, bilateral, or repetitive [72,73]. However, the same investigations report reduced sensitivity for focal, low-frequency, or anatomically restricted discharges—especially those arising from parasagittal or midline regions—underscoring the need for clinical correlation and cautious interpretation of negative or equivocal POC-EEG findings [72,73]. Nevertheless, the EEG patterns most commonly encountered in critically ill populations typically fall within the spatial and temporal resolution capabilities of modern POC-EEG platforms.

In this context, the 2HELPS2B score merits particular attention as a framework for efficient EEG triage. Developed by Struck et al., the 2HELPS2B score was derived from a multicenter retrospective cohort of 2111 patients undergoing cEEG for seizure risk assessment [74]. The model incorporates EEG features obtainable within the first hour of recording—namely sporadic epileptiform discharges, periodic or rhythmic patterns exceeding 2 Hz, lateralized abnormalities, and brief ictal discharges—along with a prior seizure history to estimate the probability of subsequent electrographic seizures. In patients without prior seizures and lacking epileptiform features during the initial hour (low 2HELPS2B score), a 1 h EEG was sufficient as a screening tool, whereas scores ≥2 supported the continuation of monitoring for at least 24 h [74].

The 2HELPS2B score is particularly relevant in ICU environments, where rapid risk stratification and judicious allocation of EEG resources are essential. Because the score relies on early EEG characteristics—many of which are readily detectable using contemporary POC-EEG devices—it may facilitate timely decision-making regarding escalation to prolonged monitoring. Although the model has thus far been validated exclusively using full-montage EEG, ongoing advances in POC-EEG hardware and signal processing raise the possibility that structured risk stratification tools such as 2HELPS2B may be adapted for reduced-montage systems in future studies [74].

## 6. Outcomes and Economic Considerations

### 6.1. Workflow Outcomes and Diagnostic Access

In a comprehensive review, Fratangelo et al. analyzed 69 studies evaluating POC-EEG systems across a broad spectrum of acute neurological conditions, including NCSE, TBI, acute ischemic stroke, delirium, and post-cardiac arrest encephalopathy [21]. Across multiple clinical contexts, the review demonstrated that quantitative EEG (qEEG) metrics derived from POC-EEG reliably reflect underlying cerebral injury. In patients with TBI and concussion, qEEG features—including spectral power asymmetries, theta–alpha ratios, and slowed dominant frequencies—were shown to correlate with structural brain lesions and align with abnormalities detected on head CT imaging [21]. Importantly, these electrophysiological markers facilitated early triage and risk stratification, enabling selective reduction in neuroimaging utilization without compromising patient safety, particularly in resource-constrained or prehospital settings. These findings reflect process-level diagnostic efficiencies, rather than direct evidence of improved neurological outcomes.

### 6.2. Impact on Clinical Decision-Making (Intermediate Outcomes)

POC-EEG signatures characterized by increased delta power, reduced alpha variability, and disrupted posterior dominant rhythm were consistently associated with delirium across ED and ICU cohorts. Integration of automated feature extraction and AI-assisted interpretation significantly improved sensitivity for delirium detection when compared with routine bedside screening tools, particularly for hypoactive presentations that are frequently missed by clinical observation alone [21,75,76]. These findings support the role of POC-EEG as an objective adjunct for early identification of acute brain dysfunction.

Continuous or repeated bedside EEG monitoring enables timely detection of malignant EEG patterns—such as generalized suppression, periodic discharges, and burst suppression—that are strongly associated with poor neurological prognosis. Additionally, quantitative measures of EEG reactivity and background continuity obtained through POC-EEG demonstrated prognostic value independent of sedative exposure, thereby informing earlier discussions regarding goals of care and therapeutic intensity [21]. These observations are concordant with international neuroprognostication guidelines, which emphasize the utility of EEG features obtained within 24–48 h after return of spontaneous circulation for predicting long-term neurological outcomes in comatose cardiac arrest survivors [76,77]. Importantly, these associations represent decision-support and prognostic utility, not evidence that POC-EEG use itself improves neurological outcomes.

### 6.3. Patient-Centered Outcomes

To date, evidence directly linking POC-EEG use to improvements in patient-centered outcomes, such as mortality, long-term functional status, or neurological recovery, remains limited and largely indirect. While EEG-derived features provide valuable prognostic information and guide clinical management, causal outcome benefit attributable to POC-EEG has not yet been conclusively demonstrated, and large randomized controlled trials evaluating patient outcomes are currently lacking.

### 6.4. Economic and Operational Outcomes

The integration of POC-EEG into acute clinical workflows has demonstrated measurable economic advantages, particularly in environments constrained by limited neurodiagnostic resources or time-sensitive clinical decision-making. These benefits arise primarily from earlier identification of NCS and NCSE, conditions that are frequently underdiagnosed in critically ill patients and are associated with prolonged ICU stays and increased healthcare utilization [78]. Timely detection through bedside EEG enables earlier therapeutic intervention, reduces secondary neurological injury, and may prevent escalation to more resource-intensive levels of care [78].

From an operational perspective, multiple studies have demonstrated that POC-EEG availability is associated with reductions in hospital length of stay (LOS). Large retrospective and prospective evaluations report a decrease in total inpatient LOS from approximately 7.4 to 6.1 days, with a corresponding reduction in ICU length of stay of 0.4–0.5 days. These improvements translate into estimated per-patient cost savings ranging from $3971 to $5633, driven largely by decreased ICU bed utilization and earlier clinical decision-making [79]. In multicenter analyses evaluating Ceribell^®^ implementation, average LOS reductions of up to three days were observed, corresponding to approximately $4850 saved per patient, in addition to increased Medicare reimbursements averaging $7300 related to improved diagnostic coding and resource alignment [33]. Collectively, these findings indicate that early neurophysiological assessment mitigates diagnostic delays that frequently prolong ICU and inpatient admissions, but should be interpreted as associative rather than casual [78].

POC-EEG implementation also reduces expenditures associated with inter-hospital transfers for emergent EEG evaluation. In settings lacking continuous EEG availability, delayed access often necessitates patient transfer to tertiary centers, incurring substantial transport and opportunity costs. Studies evaluating POC-EEG deployment report a 45% reduction in EEG-related inter-facility transfers, resulting in annual cost savings of up to $37,000. These estimates exclude indirect benefits such as reduced transfer-associated risks, expedited diagnosis, and improved continuity of care [78].

Although upfront costs are required for POC-EEG deployment, economic modeling suggests favorable cost–benefit ratios. Fixed annual deployment costs for POC-EEG systems in multi-hospital networks are estimated at approximately $120,000; however, break-even analyses indicate that as few as eight uses per month are sufficient to offset these expenditures. Compared with conventional EEG infrastructure—which requires dedicated equipment, on-site technologists, and substantial logistical coordination—POC-EEG represents a more scalable and resource-efficient model for EEG access [13].

Further cost savings accrue through reduced reliance on conventional EEG technologist staffing, particularly during nights and weekends when labor premiums are highest. Traditional EEG technologist call-ins during off-hours are estimated to generate costs of $600–$900 per study. In contrast, POC-EEG systems enable bedside application by non-EEG clinicians, such as ICU nurses or emergency physicians, with remote interpretation by neurologists or algorithm-assisted triage, effectively eliminating many of these labor-related expenses [80].

Finally, economic efficiency is enhanced through task shifting and workflow optimization. Simplified EEG hardware combined with automated seizure detection and quantitative analytics allows non-specialists to perform rapid setup and preliminary assessment. Economic modeling studies estimate up to a 25% reduction in overall hospital costs following POC-EEG implementation, attributable to shorter ICU stays, decreased exposure to anesthetic or antiseizure medications, and fewer invasive interventions such as intubation [80]. By enabling immediate data acquisition and remote interpretation, POC-EEG reduces bottlenecks in neurodiagnostic care while preserving diagnostic accuracy and responsiveness. While these workflow efficiencies are compelling, their downstream impact on patient morbidity and mortality remains to be established.

## 7. Ethical Considerations

The clinical adoption of POC-EEG introduces several ethical considerations that warrant careful evaluation as this technology becomes more widely integrated into acute neurological care. A central ethical concern relates to equity of access. Uneven availability of POC-EEG across healthcare systems—particularly between tertiary academic centers and under-resourced community hospitals—risks exacerbating existing disparities in the diagnosis and management of neurological emergencies. Given the established morbidity associated with delayed recognition of NCS and NCSE, limited access to POC-EEG capabilities may disproportionately affect critically ill and underserved populations. Ethical deployment therefore requires institutional and policy-level efforts to promote equitable distribution of POC-EEG resources across diverse clinical settings [78].

The ability of POC-EEG to rapidly detect electrographic abnormalities also imposes a duty of appropriate clinical response. Early identification of seizure activity creates a moral obligation to act in a timely and evidence-based manner; however, this obligation must be balanced against the limitations of reduced-montage EEG systems. Over-reliance on preliminary POC-EEG interpretations, particularly when performed by non-specialists or in the absence of confirmatory full-montage EEG, may increase the risk of misdiagnosis, overtreatment, or inappropriate escalation of care. Such risks underscore the ethical necessity of standardized training, clear escalation protocols, and defined thresholds for confirmatory testing to ensure that POC-EEG findings are interpreted within an appropriate clinical context [81].

Informed consent and patient autonomy represent additional ethical challenges, particularly in emergency and critical care settings where patients frequently lack decision-making capacity. While POC-EEG is noninvasive and generally considered low risk, its use involves the acquisition, storage, and remote transmission of neurophysiological data. These processes raise concerns related to data privacy, cybersecurity, and secondary use of sensitive neurological information. Ethical implementation of POC-EEG therefore requires compliance with data protection standards and transparent institutional policies governing data handling, particularly when cloud-based platforms or remote interpretation models are utilized [82,83].

The expansion of remote EEG interpretation workflows further raises ethical questions regarding the quality and comprehensiveness of neurological care. While remote review enables rapid access to expert interpretation and improves coverage in resource-limited settings, it may also reduce opportunities for in-person neurological examination and bedside clinical correlation. Ensuring that POC-EEG supplements—not substitutes—direct patient evaluation is essential to maintaining diagnostic integrity and patient-centered care.

To integrate POC-EEG ethically into clinical practice, healthcare institutions must establish robust governance frameworks that address equitable access, interpretive reliability, clinician training, informed consent, and data security. Such frameworks should be aligned with principles of beneficence, non-maleficence, justice, and respect for autonomy [84]. When implemented with appropriate safeguards, POC-EEG has the potential to enhance neurological care while adhering to fundamental ethical standards [78].

## 8. Limitations of POC-EEG

Despite its expanding clinical utility, POC-EEG presents important limitations that must be considered when interpreting findings and integrating results into clinical decision-making. A principal constraint is reduced spatial resolution, as most commercially available systems (e.g., Ceribell^®^) employ fewer electrodes than the international 10–20 EEG standard. Consequently, electrographic activity arising from parasagittal or midline cortical regions may be under-sampled, raising concern for missed focal seizure detection in select cases.

Available adult data suggest that purely parasagittal or midline seizures are relatively uncommon outside pediatric populations. In a retrospective cohort of 300 adults undergoing EEG evaluation in ED, inpatient, and intensive care settings, Gururangan et al. reported that only 2 of 17 seizure events originated from parasagittal or midline regions. Notably, in both cases, seizure activity remained visible within temporal channels, and all affected patients demonstrated overt clinical semiology—including focal motor activity, automatisms, or language disturbance—which prompted admission to non-ICU settings rather than reliance solely on electrographic screening [42]. These findings suggest that while reduced spatial coverage may theoretically limit detection, clinically significant adult seizures are often captured by reduced-montage systems.

In contrast, sporadic interictal epileptiform activity (IEA) poses a greater diagnostic challenge for POC-EEG due to its lower frequency, limited spatial distribution, and brief duration. In a retrospective comparison of Ceribell^®^ POC-EEG and standard video EEG in 20 hospitalized patients, Freund et al. demonstrated reduced sensitivity for IEA detection when discharges were infrequent or spatially restricted. One patient with isolated parasagittal discharges was not identified on POC-EEG. The authors emphasized that a nondiagnostic POC-EEG should not be interpreted as definitive exclusion of epileptiform pathology and recommended confirmatory conventional EEG—particularly when clinical suspicion remains high or antiseizure medication decisions depend on electrographic findings [72].

Additional limitations arise from the use of automated seizure detection algorithms, which are designed to assist rapid clinical triage but are not substitutes for expert EEG interpretation. While contemporary algorithms demonstrate strong performance for status epilepticus detection, they are optimized for seizure burden rather than isolated or brief events. In a large multicenter evaluation of 665 recordings across 11 hospitals, Kamousi et al. (2024) [25] reported that Clarity version 6 achieved high sensitivity (95%) and specificity (97%) for detecting status epilepticus at a seizure burden threshold of 90%, with a positive predictive value of 53% and a negative predictive value of 99% [25]. These results support the algorithm’s utility for identifying high-risk patients but underscore dependence on seizure burden thresholds [25].

However, false-negative results remain clinically relevant. In a retrospective review of 21 comatose post-cardiac arrest patients monitored with Ceribell^®^, Villamar et al. found that 4 patients exhibited electrographic seizures—two meeting criteria for status epilepticus—on expert review of raw EEG data, despite Clarity version 4 reporting 0% seizure burden in all four cases [85]. Responding to these findings, Parvizi et al. emphasized that the Clarity algorithm is intended to alert non-expert clinicians to the likelihood of impending or sustained status epilepticus rather than to exhaustively identify all seizure events. They further noted that post-cardiac arrest patients represent a technically challenging population due to myogenic activity, generalized slowing, and other signal artifacts that limit algorithmic performance [29,85]

Algorithm performance also varies according to seizure burden thresholds. In an earlier retrospective study of 353 adult patients, Kamousi et al. [25] demonstrated that lowering the seizure burden threshold from 50% to 10% increased sensitivity for seizure detection from 88% to 100% but decreased specificity from 82% to 60%. Only two seizures identified by expert reviewers were missed by Clarity, both lasting less than 30 s. During the study period, the system generated a false-positive rate of approximately 0.36 events per hour [25]. These findings highlight the persistent vulnerability of automated systems to brief or low-burden seizures and reinforce the necessity of timely expert interpretation of raw EEG data, particularly when clinical decisions carry significant therapeutic or prognostic consequences.

Regulatory clearance of POC-EEG devices and associated analytic software reflects demonstration of safety and defined performance characteristics under specific conditions. Clearance should not be interpreted as evidence of clinical superiority, nor does it obviate the need for expert EEG review or confirmatory conventional EEG in appropriate clinical contexts. Importantly, performance characteristics, regulatory status, and workflow advantages vary substantially among devices, and comparative effectiveness data between POC-EEG platforms remain limited.

## 9. Future Studies

Future research should prioritize rigorous evaluation of the diagnostic performance and clinical impact of automated interpretation tools integrated within point-of-care EEG (POC-EEG) systems, including the Clarity AI platform. Although these systems demonstrate strong performance for high seizure burden and status epilepticus detection, reduced sensitivity has been reported for seizures arising from parasagittal or midline regions and in patients with hypoxic–ischemic encephalopathy following cardiac arrest [85,86]. Prospective, externally validated studies across heterogeneous patient populations are required to define algorithmic limitations, optimize seizure burden thresholds, and establish best-practice workflows for combining automated outputs with expert human review.

Another critical area for future investigation is the differentiation of epileptic seizures from psychogenic non-epileptic events (PNES) and related seizure mimics in emergency and acute care environments. Prior studies demonstrate that diagnostic accuracy for seizure-like events remains limited, with reported sensitivities of approximately 73% among neurologists and as low as 44% among emergency medicine physicians when relying on clinical assessment alone [87]. Reduced-montage EEG systems may offer objective neurophysiological data that improve early diagnostic discrimination; however, false-negative findings and artifact-related ambiguities persist. Prospective studies should assess whether early POC-EEG deployment improves diagnostic accuracy for PNES and acute symptomatic non-epileptic events, particularly when combined with standardized clinical algorithms.

Closely related is the challenge of distinguishing post-ictal coma from ongoing nonconvulsive status epilepticus (NCSE). Early administration of sedatives and antiseizure medications in patients with transient post-ictal encephalopathy may contribute to unnecessary intubation, prolonged mechanical ventilation, and avoidable ICU admissions. Early placement of POC-EEG has potential utility as a rapid rule-out tool for NCSE, enabling de-escalation of treatment in appropriate patients. Controlled studies examining whether early EEG-guided decision-making reduces unnecessary pharmacologic escalation and ICU utilization are needed [20].

Given the high incidence of NCSE among critically ill patients, future studies should also evaluate whether earlier initiation of POC-EEG monitoring in the ICU translates into improved clinical outcomes. Endpoints of interest include reductions in time to seizure diagnosis, duration of electrographic seizure activity, length of ICU stay, in-hospital mortality, and long-term neurological outcomes. Randomized or pragmatic comparative-effectiveness trials could clarify whether early EEG-guided intervention alters the disease trajectory in high-risk populations, including patients with sepsis, intracerebral hemorrhage, and traumatic brain injury.

Finally, continued expansion of POC-EEG technology into the prehospital setting represents an important frontier. Feasibility studies have demonstrated that POC-EEG acquisition by emergency medical services (EMS) personnel is possible; however, outcome-focused data remain limited. Future investigations should explore whether prehospital POC-EEG-guided management enables earlier identification and treatment of status epilepticus, reduces unnecessary hospital and ICU admissions, and improves survival or functional outcomes. Such studies will be essential to determine whether the theoretical temporal advantage of prehospital EEG translates into measurable clinical and system-level benefits.

In parallel with ongoing technological and algorithmic development, future investigations should prioritize the translation of POC-EEG into clear, clinically actionable workflows suitable for time-pressured acute care environments. Despite growing evidence supporting diagnostic and operational benefit, variability in clinician familiarity, interpretation confidence, and deployment thresholds continues to limit consistent utilization. Pragmatic studies evaluating standardized implementation strategies—including clearly defined indications where POC-EEG is sufficient for triage, structured escalation criteria prompting conventional EEG, and concise clinical “red-flag” scenarios—are needed to optimize uptake and promote diagnostic stewardship. Key practical considerations relevant to bedside decision-making are summarized in Table 3, highlighting domains where POC-EEG is most effective, scenarios where caution is warranted, and circumstances requiring confirmatory conventional EEG. Aligning future research with these real-world usability considerations will be essential to ensure that POC-EEG not only accelerates diagnosis but also improves clinical effectiveness, safety, and equity across diverse acute neurological care settings.

## 10. Conclusions

POC-EEG represents a significant advancement in the timely diagnosis and management of NCS and NCSE, particularly in emergency and critical care settings. This review highlights the growing body of evidence supporting its clinical utility across a range of neurological conditions, including stroke, traumatic brain injury, delirium, and post-cardiac arrest encephalopathy. POC-EEG systems offer practical advantages, including rapid deployment, cost-effectiveness, and increased accessibility, particularly in resource-limited environments.

The integration of artificial intelligence and simplified hardware has further expanded the reach of EEG monitoring, enabling non-specialists to contribute to early seizure detection and treatment decisions. However, limitations remain, including reduced spatial resolution, susceptibility to artifacts, and the need for cautious interpretation of automated outputs. These challenges underscore the importance of combining POC-EEG findings with clinical judgment and, when necessary, follow-up with conventional EEG.

Future research should focus on validating diagnostic algorithms, optimizing device sensitivity and specificity, and evaluating long-term outcomes associated with POC-EEG-guided interventions. As technology continues to evolve, its role in acute neurological care is likely to expand, offering a scalable and impactful solution to enhance diagnostic accuracy and improve patient outcomes in diverse clinical settings.

## Figures and Tables

**Table 2:** Technical and clinical characteristics of selected point-of-care EEG (POC-EEG) systems reported in the literature.

Device	Electrodes	EEG Montage	Regulatory Clearance	Technical Features	Reported Limitations	Setup and Workflow Characteristics
Ceribell Manufacturer: CeriBell, Inc. City: Sunnyvale, CA Country: USA	10	8 bipolar pairs	FDA-cleared EEG acquisition device	AI seizure burden display Cloud based data CT compatible	Limited spatial coverage with potential to miss focal or parasagittal seizures; false-positive detections reported; AI outputs require clinical correlation; not MRI compatible	Rapid bedside setup; designed for use by non-neurologist clinicians in ED and ICU settings
ZetoEEG Manufacturer: Zeto, Inc. City: Santa Clara, CA Country: USA	21	Limited montage	FDA-cleared EEG acquisition device	Wireless EEG with integrated video; dry soft-tip electrodes; automated seizure-detection outputs reported in selected studies	Potential for false-positive detections and missed seizures; not MRI compatible	Portable, wearable headset; dry electrodes without gel or skin preparation; rapid deployment
BrainScope Manufacturer: BrainScope Company, Inc. City: Bethesda, MD Country: USA	8	Frontal montage	FDA-cleared EEG acquisition device	Quantitative EEG-based indices for structural injury and brain function assessment; real-time waveform display	Not intended as a substitute for CT imaging; no seizure-specific AI detection; transient scalp discomfort reported	Handheld, battery-powered, wireless device; touchscreen interface
CerebAir AE-120 Manufacturer: Nihon Kohden Corporation City: Tokyo Country: Japan	8	Reduced montage	FDA-cleared EEG electrode system (used as predicate device)	Lightweight, wireless EEG headset; emphasis on signal quality and ease of application	No automated seizure-detection software; electrode-impedance challenges reported; skin irritation with prolonged use reactions	Minimal controls; rapid application; designed to integrate with external EEG systems
BrainWatch (from Natus) Manufacturer: Natus Medical Incorporated City: Middleton, WI Country: USA	10	Limited montage	FDA-cleared EEG acquisition device	Integration with commercial EEG platforms; optional AI-assisted seizure-detection algorithms; remote data review	False-positive detections and missed seizures reported; AI outputs require expert interpretation	Wearable wireless system; tablet-based interface; integration with hospital EEG infrastructure
OpenBCI Manufacturer: OpenBCI, Inc. City: Brooklyn, NY Country: USA	8–16	8–16 channels (depending on which bioamplifier it is paired with))	No FDA clearance	Open-source biosignal acquisition (EEG/EOG/EMG); customizable hardware and software	Not designed for clinical diagnosis; no integrated seizure-detection software; variable fit and signal quality	Rapid setup; wireless (Bluetooth); primarily research-oriented
BrainHealth Manufacturer: Rhythmlink International, LLC City: Columbia, SC Country: USA	10	Headset only (no amplifier)	FDA-cleared EEG accessory	EEG headset compatible with CT; MRI-compatible electrode options reported	Requires connection to a separate EEG acquisition system; no automated seizure-detection software	Short setup time; wireless, wearable headset

This table provides a descriptive comparison of selected point-of-care EEG (POC-EEG) systems based on published technical specifications and peer-reviewed clinical studies. The table focuses on EEG configuration, signal acquisition, and reported clinical use cases rather than product performance claims.

**Table 3:** Ten Key Takeaways for Clinicians Using POC-EEG.

Key Takeaway	Why It Matters Clinically
POC-EEG enables rapid detection of NCSE and high seizure burden	Setup in minutes allows earlier treatment in patients with unexplained altered mental status
2. POC-EEG is best for detecting sustained or generalized cerebral activity	High sensitivity for NCSE and post-convulsive NCSE; lower sensitivity for focal or brief events
3. Negative POC-EEG does not definitively exclude seizures	Focal, parasagittal, or low-burden seizures may be missed
4. POC-EEG should be viewed as a triage tool, not a replacement for cEEG	Helps rule in high-risk pathology and prioritize escalation
5. AI seizure alerts are reliable for high seizure burden but limited for isolated events	Automated outputs must be interpreted in clinical context
6. POC-EEG is particularly useful in community and resource-limited hospitals	Reduces unnecessary transfers and delays to diagnosis
7. Artifact is common and must be actively assessed	Movement, EMG, and dry electrodes can mimic seizures
8. Early POC-EEG findings often prompt meaningful treatment changes	Supports escalation and de-escalation of antiseizure therapy
9. Certain malignant EEG patterns (e.g., burst suppression) are reliably detected	Valuable for post-cardiac arrest prognostication and triage
10. Timely expert review remains essential when results are equivocal	Raw EEG review improves diagnostic accuracy and prevents overtreatment

## Data Availability

All the data are presented in the manuscript.

## Abbreviations

The following abbreviations are used in this manuscript:

ACNS	American Clinical Neurophysiology Society
AI	Artificial Intelligence
BCI	Brain–Computer Interface
cEEG	Continuous Electroencephalography
CMS	Centers for Medicare & Medicaid Services
CT	Computed Tomography
ED	Emergency Department
EEG	Electroencephalography
ERP	Event-Related Potential
FDA	Food and Drug Administration
fEMG	Facial Electromyography
fNIRS	Functional Near-Infrared Spectroscopy
GBD	Global Burden of Disease
ICU	Intensive Care Unit
IEA	Interictal Epileptiform Activity
ILAE	International League Against Epilepsy
LOC	Loss of Consciousness
MRI	Magnetic Resonance Imaging
mTBI	Mild Traumatic Brain Injury
NCS	Non-Convulsive Seizures
NCSE	Non-Convulsive Status Epilepticus
NPV	Negative Predictive Value
POC-EEG	Point-of-Care Electroencephalography
PPV	Positive Predictive Value
ROC	Receiver Operating Characteristic
SE	Status Epilepticus
SzB	Seizure Burden
TBI	Traumatic Brain Injury
